# Does intramedullary nail have advantages over dynamic hip screw for the treatment of AO/OTA31A1-A3? A meta-analysis

**DOI:** 10.1186/s12891-023-06715-0

**Published:** 2023-07-18

**Authors:** Fei Yu, Yang-Wei Tang, Ju Wang, Zhi-Cheng Lin, Yu-Bin Liu

**Affiliations:** 1 College of Management, Guangdong Polytechnic Normal University, Guangzhou, China; 2 Department of Orthopedics, Huaiji People’s Hospital, Zhaoqing, China; 3 Orthopedics Center, Department of Orthopedics and Traumatology, Zhujiang Hospital, Southern Medical University, No.253. Gongye Middle Avenue, Haizhu District, Guangzhou, Guangdong 510280 China

**Keywords:** Intramedullary nail, Dynamic hip screw, Hip fractures, meta-analysis

## Abstract

**Background:**

Hip fractures are still unsolved problems nowadays. We evaluated the functional outcomes and complications in the treatment of hip fractures (AO/OTA31A1-A3) to find potential difference and risk between intramedullary nail (IMN) and dynamic hip screw (DHS).

**Method:**

We searched PubMed, Embase, Cochrane library up to 19 June 2023 and retrieved any studies comparing IMN and DHS in treatment of Hip fractures. The main outcomes and complications were extracted from the included studies. The fixed-effect model was selected to pool the data for homogeneous studies (I^2^ < 50%). Otherwise, the random effects model was selected (heterogeneity, I^2^ > 50%). The analysis of sensitivity and subgroup was performed to explore the homogeneous studies among studies. The *p*-value of less than 0.05 was considered statistically significant.

**Results:**

30 RCT studies were included in this meta-analysis. There were significant difference of in the items of blood loss, screening time, femoral neck shortening, non-union, and femoral fractures (*p* < 0.05). Significant difference was found in the parameter of open reduction of fracture after sensitive analysis (*p* < 0.05). No significant difference was found in the parameter of Mobility Score at the last follow-up after sensitive analysis (*p* ≥ 0.05). There was no significant difference in the parameters of open reduction of fracture, required blood transfusion, mean surgical time, hospital stays, time to healing, mean Harris Hip Score, infection, cut out, poor reduction, breakage of implant, failure of fixation, reoperation, and systemic complications of chest infection, decubital ulcer, urinary tract infection and persistent pain in the hip (*p* ≥ 0.05).

**Conclusions:**

Our meta-analysis revealed that hip fractures treated with IMN have merits with lower rate of blood loss, femoral neck shortening and non-union; shortcoming of increased risk of femoral fractures. It is suggested that special attention should be paid to the risk of femoral fracture when intramedullary nail was inserted in the intraoperative.

## Background

Hip fractures are becoming increasingly common as population ages. The incidence of hip fractures is expected to reach 2.6 million by 2025 and 4.5 million by 2050 [1]. These fractures cause significant morbidity and increased mortality [2], 12–17% of patients with a hip fracture died within the first year, especially those elderly with limited activity [3]. Hip fractures include femoral neck fracture and intertrochanteric facture, and the most common fracture classification was the AO system. The choice of surgical treatment is the best strategy for the hip fractures, which has advantage of early rehabilitation and functional recovery, and reduces the risk of postoperative complications [4]. Published papers supported that the indication of dynamic hip screw (DHS) was applied for the treatment of stable fractures, basicervical fractures, and trans-cervical fractures, while intramedullary nail (IMN) was used for the treatment of stable fractures, unstable fractures, per trochanteric, reduced lateral wall thickness, reverse obliquity unstable type fractures [5–7].

**Fig. 1:**
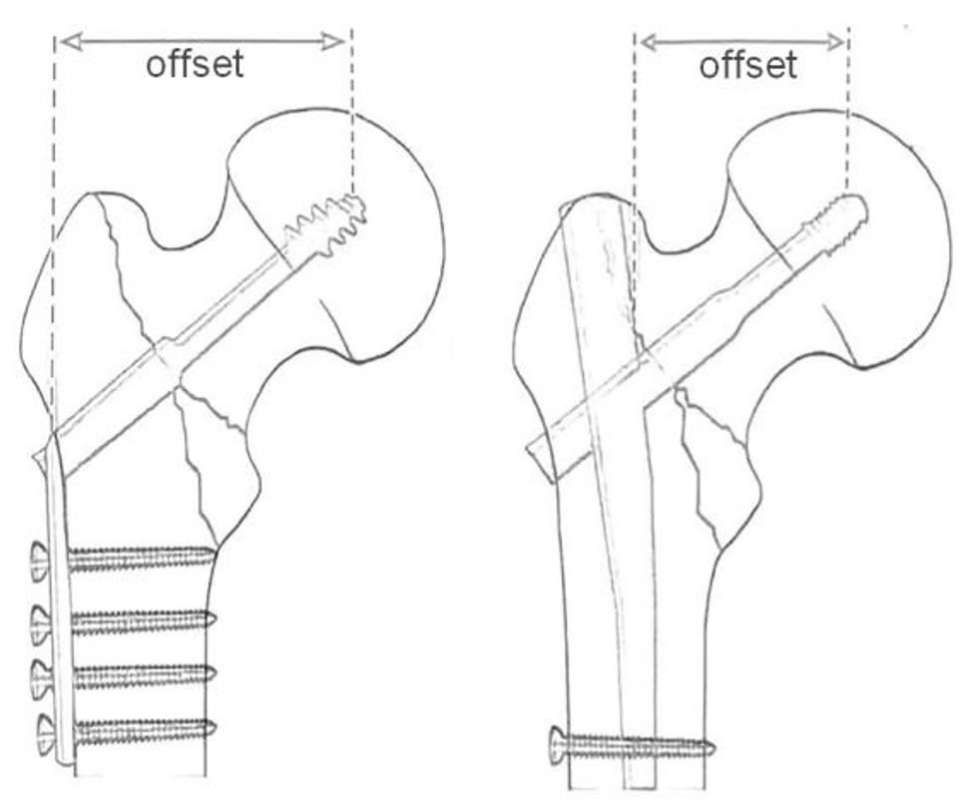
Smaller offset in intramedullary nail

The biomechanical superiority of IMN was that the offset was small for the reason of the femoral shaft axis nearer to the center of rotation of the hip (Fig. 1), resulting in a shorter lever arm and lower bending moment on the device [8, 9]. The characteristics of sliding and compressing of IMN can promote the healing of the fracture end [10]. The intrinsic mechanical solidity and load distribution allows nails to support most of the forces acting on the hip during gait (axial weight bearing and bending moments) avoiding stress on the fracture site [11]. In addition, a telescoping displacement of the proximal fragment was prevented by the main nail when the failure of initial stability occurred [8, 12]. On the other hand, DHS implies a relative instability in this system, not only axially but also transversally and in rotation. This potential instability could adversely affect the functional outcome, pain and healing for the hip fractures [13]. Theoretically, the fixation of IMN predicts higher healing and less complication for hip fractures. However, the inconsistent results were reported for hip fractures including functional outcome, blood loss, surgical time, complications, reoperations and so on [14–17]. The 2023 meta-analysis by Zhang et al. [18] revealed that PFNA exhibited a beneficial role in Harris Scores, operation time, blood loss, hospital stay, healing time, cut-out, reoperation, union problems, and infection; however, DHS was superior to PFNA in hidden blood loss, postoperation drainage, total blood loss, and femoral shaft fracture. Another 2022 meta-analysis by Wessels et al. [19] reported that no significant difference was found in the complication of nonunion, infection, and mortality when (AO/OTA) 31A1-A3 fractures treated with either DHS or IMN. A 2022 meta-analysis by Xu et al. reported that PFN had shorter operative time and led to less intraoperative blood loss, no difference was seen between PFN and DHS for non-union, risk of implant failure and revision surgery [20]. Given these controversial results, we remained skeptical of the relevant conclusions of these studies. We think there are many common features for the intramedullary nailing of PFNA, PFN, and GN for the treatment of hip fractures. The major difference between IMN and DHS was the offset in the device design (Fig. 1). In present meta-analysis, we considered the IMN devices including PFNA, PFN, INTERTAN nail, and GN as the same type of internal fixation device for hip fractures. The aim of present study was to systemically assess the relative parameters difference in the process of intra-operation, post-operation and complications between two groups of IMN versus DHS in treating hip fractures.

**Fig. 2:**
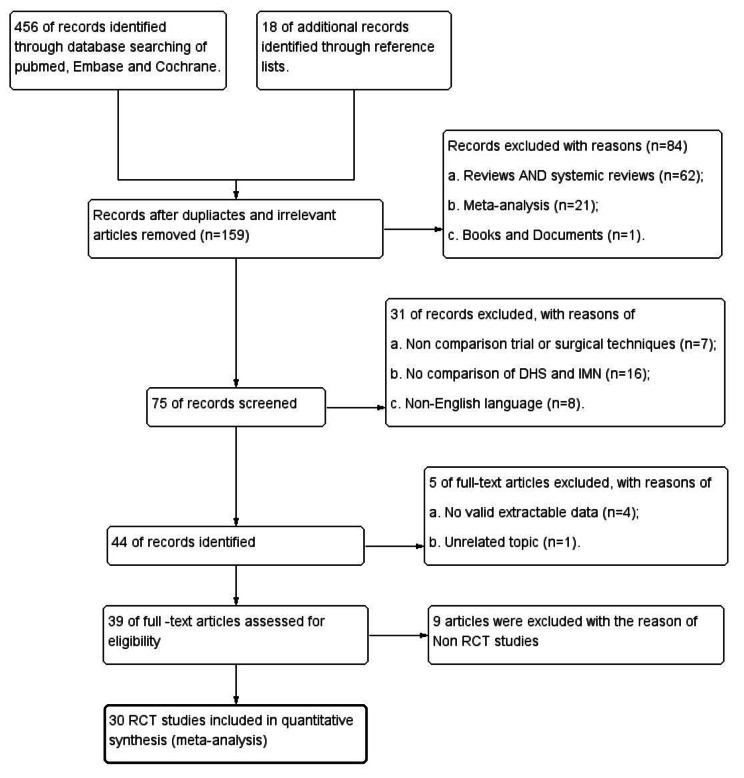
Flowchart of study selection

## Materials and methods

### Search strategy

In this study, we defined hip fractures as AO/OTA31A1-A3 to generalize all fracture types of the proximal femur. We searched the electronic databases of PubMed, Cochrane, and Embase on 19 June 2023 for all published literature. The following search terms were identified: ((Intramedullary Fracture Fixation) OR (Intramedullary Nailing)) AND ((Dynamic hip screw) OR (Sliding hip screw)) AND ((Hip Fractures) OR (Trochanteric Fractures) OR (Femoral neck fracture)). In addition, we conducted a hand search of reference lists from the eligible studies to prevent any omissions.

### Inclusion and exclusion criteria

The inclusion criteria were identified as follow: (1) randomized controlled trial (RCT) studies; (2) Hip fractures (AO/OTA: A1-A3); (3) the intervention included dynamic hip screw (DHS) OR sliding hip screw (SHS) and comparison included intramedullary nailing (gamma nails, proximal femoral nail (PFN), proximal femoral nail antirotation (PFNA) and Intertan nail);(4) full text paper can be retrieved; (5) follow-up was more than 4 months. Studies would be excluded for the reasons: (1) Non-RCT studies for IMN and DHS; (2) included the non-traumatic fractures, such as pathologic fractures; (3) not original articles, including biomechanical or cadaveric studies, technical notes, letters to the editor, conference abstracts, expert opinions, review articles, meta-analyses, and case reports; (4) not report results that would allow us to obtain or calculate comparative data; and (5) non-English language.

### Study selection

All studies were independently reviewed by two reviewers according to the inclusion and exclusion criteria. The full texts of all the relevant studies were obtained and reviewed. Any disagreements were resolved by consensus with another senior reviewer.

### Data extraction

Data extraction was carried out critically and independently by two researchers, while a third researcher resolved any disputes. The following data was extracted: the first author’s name, publication year, research country, study design, interventions, sample size, age, gender, type of fracture, follow-up time.

### Outcomes

The included studies were identified at least one of the following outcomes: (a) intra-operative difference (open reduction of fracture, blood loose (ml), required blood transfusion, screening time (min), mean surgical time (min)); (b) post-operative difference (hospital stay (days), time to healing (days), femoral neck shortening (mm), mean Harris hip score and mobility score); (c) total orthopedic complications; (d) subgroup analysis of orthopedic complications (infection, cut out, poor reduction, breakage of implant, non-union, femoral fracture, failure of fixation and reoperation); (e) systemic complications (chest infection, decubitus ulcer, urinary tract infection and persistent pain in the hip).

**Table 1:** The characteristic of included papers

Authors	Country	Design	Intervention	Control	Patients		Age (mean, year)		Gender (M:F)		Type of fracture	Follow-up (At least)
					IMN	DHS	IMN	DHS	IMN	DHS		
Adams 2001 [33]	UK	RCT	GN	DHS	203	197	81.2 (48–99)	80.7 (32–102)	39/164	49/148	AO/OTA: A1.1-A3.3, B2.1	12 Months
Ahrengart 2002 [38]	Sweden, Finland	RCT	GN	DHS	210	216	-	-	F:71%	F:72%	Evans: I-V	6 Months
Hoffman 1996 [34]	New Zealand	RCT	GN	DHS	31	36	83.2 ± 8.1	79.0 ± 10.4	4/27	12/24	Jensen (I-V), Stable, Unstable	6 Months
Bridle 1991 [42]	UK	RCT	GN	DHS	49	51	81	82.7	7/44	9/40	Evans	6 Months
Aune 1994 [8]	Norway	RCT	GN	DHS	177	201	82(49–96)	78(45–93)	66/109	89/114	Stable, Unstable, Subtrochanteric	17 Months
Barton 2010 [43]	UK	RCT	GN	DHS	100	110	83.1 (42 to 99)	83.3 (56 to 97)	19/81	25/85	AO/OTA: A2	1 Year
Leung 1992 [35]	Hong Kong	RCT	GN	DHS	113	113	80.86 ± 8.41	78.27 ± 9.46	25/68	30/63	Jensen Evans	6–12 Months
Hardy 1998 [44]	Belgium	RCT	GN	DHS	50	50	81.7 ± 11.8	79.5 ± 10.7	42/83	35/15	Jensen and Michaelsen	12 Months
Butt 1995 [45]	UK	RCT	GN	DHS	47	48	55–92 (mean 79)	47–101 (mean 78)	16/31	13/35	Stable, Unstable, Subtrochanteric	Untile Union
Park 1998 [36]	Korea	RCT	GN	DHS	30	30	73.7	72.2	10/20	14/16	Tronzo: II-IV	12–31 Months
Utrilla 2005 [46]	Spain	RCT	GN	DHS	104	106	80.6 ± 7.5	79.8 ± 7.3	66/38	78/28	Stable, Unstable	12 Months
Aktselis 2014 [47]	Greece	RCT	GN	DHS	36	35	82.9 ± 5.8	83.1 ± 6.5	28/8	28/7	AO/OTA: A2.2, A2.3	12 Months
Pajarinen 2005 [48]	Finland	RCT	PFN	DHS	54	54	80.9 ± 9.1	80.3 ± 10.8	13/41	14/40	AO/OTA: A1, A2	4 Months
Saudan 2002 [24]	Switzerland	RCT	PFN	DHS	100	106	83 ± 9.7	83.7 ± 10.1	24/76	22/84	AO/OTA: A1, A2	1 Year
Pajarinen 2004 [5]	Finland	RCT	PFN	DHS	24	24	78.8 ± 9.7	79.8 ± 10.2	4/20	5/19	AO/OTA: A2	4 Months
Papasimos 2005 [49]	Greece	RCT	PFN	DHS	40	40	79.4	81.4	17/23	14/26	AO/OTA: A2, A3	1 Year
Adeel 2020 [14]	Pakistan	RCT	PFN	DHS	34	34	59.32 ± 2.39	60.88 ± 12.49	25/9	22/12	AO/OTA: A2, A3	12 Months
Schemitsch 2023 [50]	INSITE Investigators	RCT	IMN	DHS	418	415	78.2 (26–102)	78.8 (18–100)	153/265	138/277	AO/OTA: A1-A2,	52 weeks
Reindl 2015 [51]	Canada	RCT	IMN	DHS	102	92	82 ± 8.6	80 ± 9.9	57/55	31/61	AO/OTA: A2	1 Year
Harrington 2002 [52]	UK	RCT	IMN	DHS	50	52	83.8 ± 8.5	82.1 ± 8.6	10/40	11/41	Evans (III-V)	12 Months
Little 2008 [53]	UK	RCT	Holland Nail	DHS	92	98	82.6 (54 to 102)	84.2 (50 to 98)	84/8	78/20	AO/OTA: A1, A2, A3	1 Year
Parker 2012 [54]	UK	RCT	Targon PF	DHS	300	300	82.4 (26–104)	81.4 (27–104)	52/248	69/231	AO/OTA: A1, A2, A3	1 Year
Parker 2017 [55]	UK	RCT	Targon PF	DHS	200	200	82.0(36–101)	83.2 (25–105)	60/140	47/153	AO/OTA: A1, A2, A3	1 Year
Matre 2013 [56]	Norway	RCT	InterTAN	DHS	341	343	84.1	84.1	83/258	88/255	AO/OTA: A1, A2, A3	1 Year
Sanders 2017 [57]	Canada	RCT	InterTAN	DHS	123	126	-	-	-	-	AO/OTA: A1, A2	12 Months
Singh 2019 [15]	India	RCT	PFNA	DHS	30	30	72.76 ± 9.5	69.33 ± 5.7	9/21	16/14	AO/OTA: A1.1-A2.1	1 Year
Huang 2017 [58]	China	RCT	PFNA	DHS	30	30	75.07 ± 7.87	74.01 ± 7.25	15/15	17/13	Tronzo–Evans (III-V)	12–24 Months
Xu 2010 [25]	China	RCT	PFNA	DHS	51	55	78.5 ± 7.97	77.9 ± 7.82	15/36	16/39	AO/OTA: A2	12 Months
Zehir 2015 [59]	Turkey	RCT	PFNA	DHS	96	102	77.22 ± 6.82	76.86 ± 6.74	37/59	39/63	AO/OTA: A2, A3	6 Months
Zou 2009 [60]	China	RCT	PFNA	DHS	58	63	65 (37–91)	65 (34–89)	M:21%	M:24%	AO/OTA: A2, A3	1 Year

Targon PF: Targon Proximal Femoral Nail; GN: Gamma Nail, PFN: Proximal Femoral Nail; PFNA: Proximal Femoral Nail with Anti-rotation; RCT: Randomized Controlled Trials; DHS: Dynamic Hip Screw

### Risk of bias assessment

Two authors independently assessed the risk of bias of all studies included in the meta-analysis. The Cochrane risk of bias (ROB) assessment tool was applied for the risk of bias assessment of RCT studies [21]. The included items were listed as follows, random sequence generation (selection bias), allocation concealment (selection bias), blinding of participants and investigators (performance bias), blinding of outcome assessment (detection bias), incomplete outcome data (attrition bias), and selective reporting (reporting bias), and other bias. Each study was classified in each domain as low, unclear, or high risk of bias. Disagreement was resolved by consensus amongst group discussion.

**Fig. 3:**
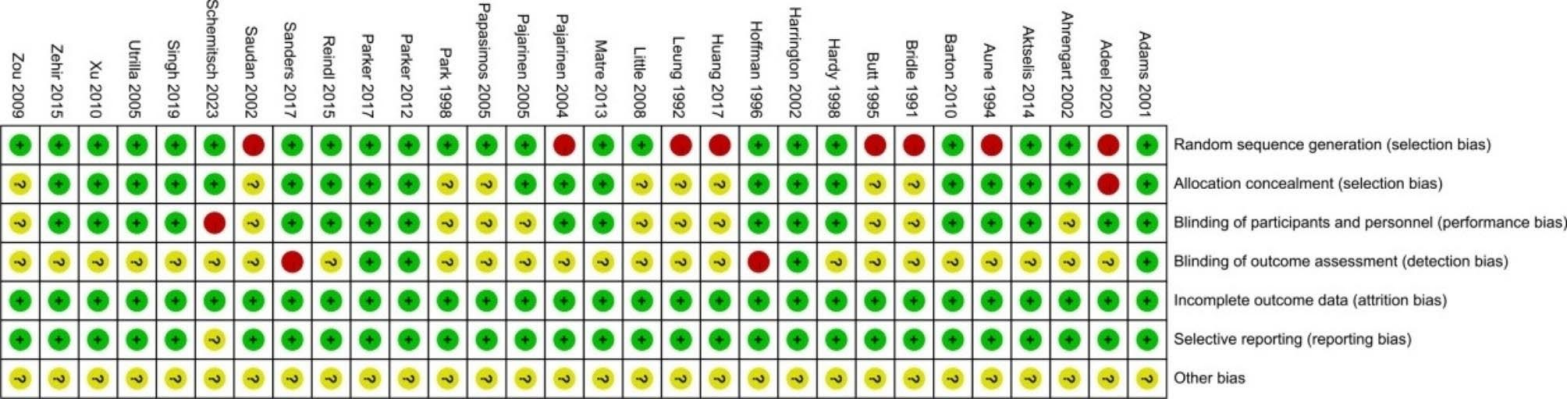
The risk of bias summary for the included studies

### Statistical analysis

Statistical analysis was performed by the software of RevMan 5.4 (The Cochrane Collaboration, 2020). We used standardized mean differences (SMD) and 95% confidence intervals (CI) to express continuous data, and the pooled odds ratio (OR) with a 95% confidence interval (CI) to calculate for dichotomous outcomes. Heterogeneity was assessed with the I^2^. If there was significant heterogeneity (I^2^ > 50%), we selected a random effects model to pool the data. On the contrary (I^2^ < 50%), the fixed-effect model was selected. A method of sensitivity analysis and subgroup analysis was performed to explore the source of heterogeneity [22]. Publication bias was investigated by funnel plot and an asymmetric plot suggested possible publication bias [23]. All *p*-values were two-sided and a *p*-value of less than 0.05 was considered statistically significant.

**Fig. 4:**
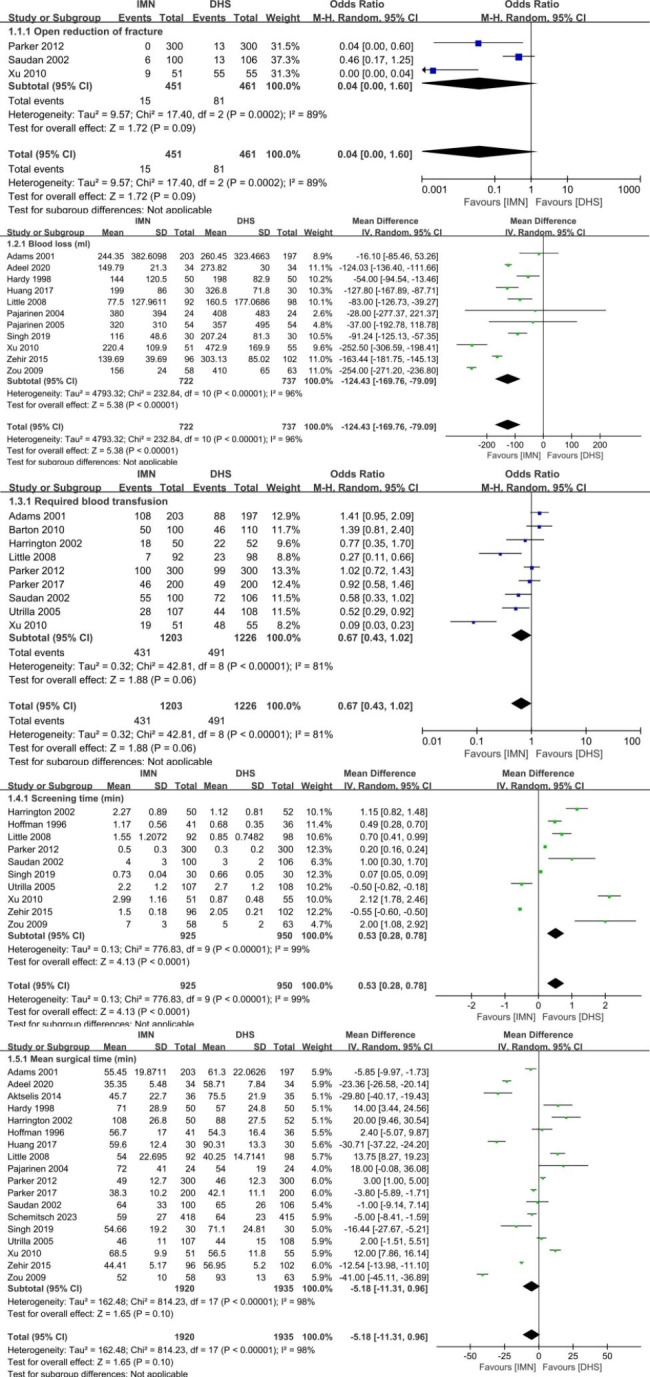
Forest plot comparing the complications in intra-operation of open reduction of fracture, blood loss, required blood transfusion, screening time and mean surgical time

## Results

### Literature characteristics

The initial search of the databases yielded 456 studies from Pubmed, Embase and Cochrane; 18 additional studies were added from the reference sources. 318 studies were removed for the reason of duplicates and unrelated topic. 84 studies were excluded for the reasons of review, meta-analysis, books and documents. Then, 31 of records were excluded with the reasons of non-comparison trial or surgical techniques, non-comparison of DHS and IMN, and non-English language. 44 remaining full-text articles were then screened, 5 studies were excluded with the reason of no valid extractable data, and then 9 articles were excluded with the reason of Non RCT studies. Finally, 30 studies were eligible for the meta-analysis. The PRISMA flow diagram and checklist for this search is shown in Fig. 2. Of the included 30 studies, a total of 3293 subjects underwent fixation with IMN and 3357 with DHS. The related characteristic of the studies was summarized in Table 1. The follow-up time for the involved studies was more than 4 months.

**Fig. 5:**
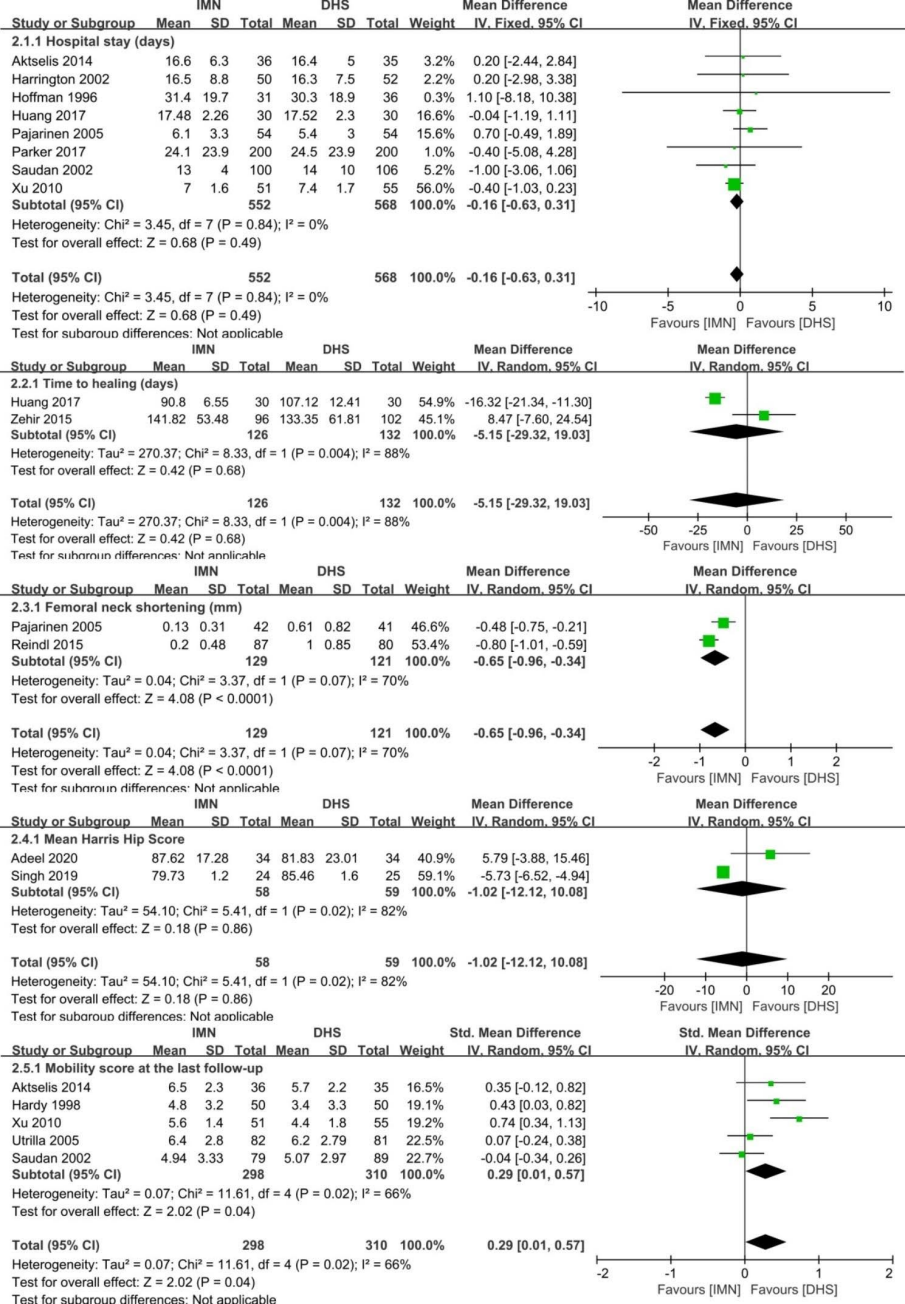
Forest plot comparing the complications in post-operation of hospital stays, time to healing, femoral neck shortening, mean Harris score and mobility score at last follow up

### Quality assessment

The included RCT studies were assessed for the risk of bias according to Cochrane Handbook for Systematic reviews and interventions [21]. For the included 30 RCT studies, 8 studies were assessed for high risk for random sequence generation. Low bias was found in terms of incomplete outcome data and selective reporting. The risk bias of each item was summarized in the Fig. 3.

**Fig. 6:**
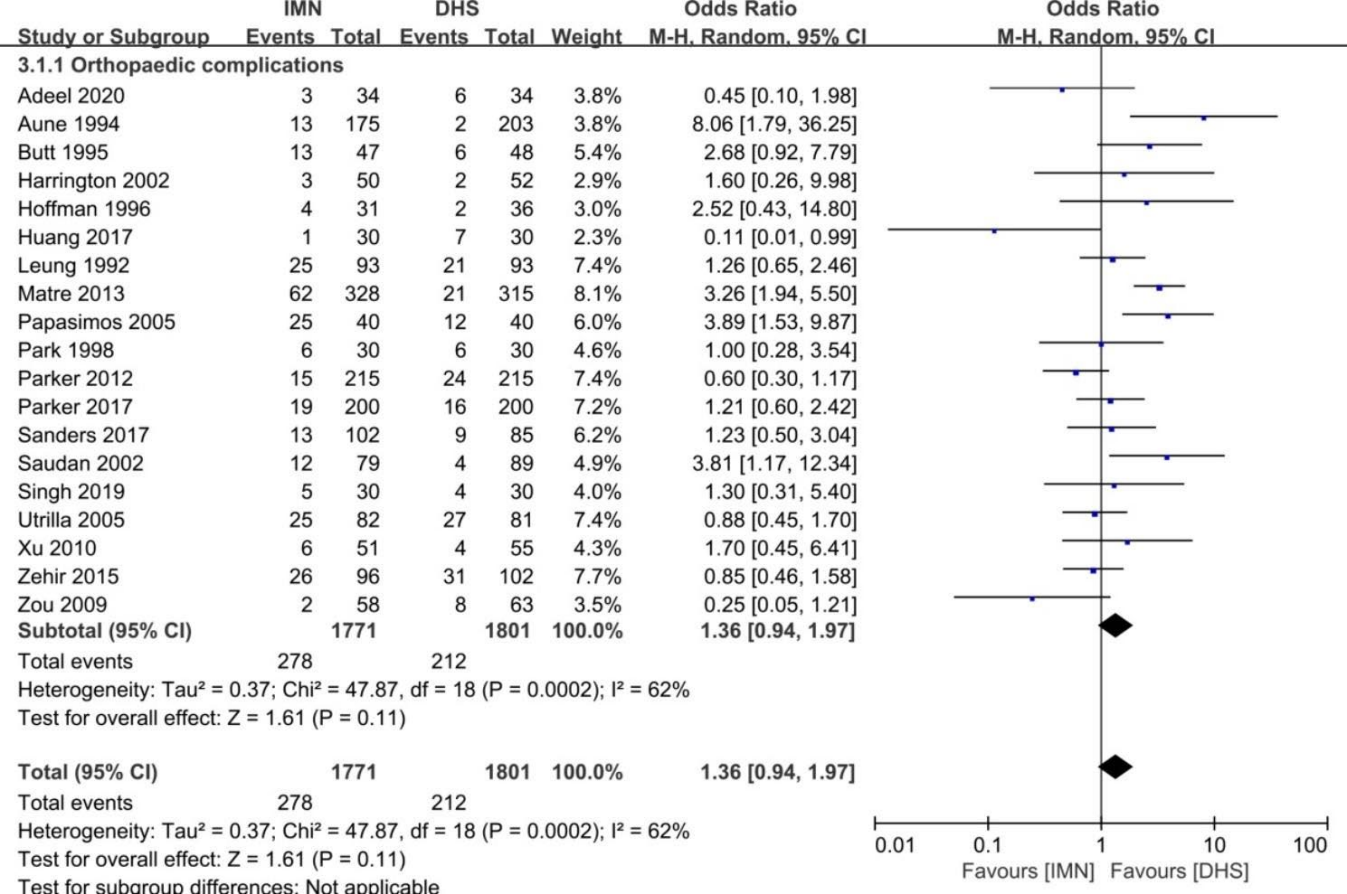
Forest plot comparing the total complications

### Outcomes

#### Intraoperative details

The operative difference between the groups of IMN and DHS was presented in the Fig. 4. Significant difference was found in the parameters of blood loss (MD=-124.43ml, 95%CI [-169.76, -79.09], *p* < 0.0001), and screening time (MD = 0.530.43 min, 95%CI [0.28, 0.78], *p* < 0.0001). There was no significant difference in the items of open reduction of fracture (OR = 0.04, 95%CI [0.00, 1.60], *p* = 0.09), required blood transfusion (OR = 0.67, 95%CI [0.43, 1.02], *p* = 0.06) and mean surgical time (OR=-5.18, 95%CI [-11.31, 0.96]). There was great heterogeneity for the parameter of open reduction of fracture (*p* = 0.0002, I^2^ = 82%). So, the sensitivity analysis was conducted by excluding Saudan et al. [24], then the remaining studies were homogeneous (*p* = 0.16, I^2^ = 49%). Significant difference was found between two groups in the parameter of open reduction of fracture (OR = 0.01, 95%CI [0.00, 0.14], *p* = 0.0009). For other parameters of blood loss, required blood transfusion, screening time and mean surgical time, the heterogeneity was inevitable between the studies after sensitivity analysis, and the random-effect model was applied to pool the data for statistical analysis. The results of operative details revealed that there were advantages of IMN in the items of blood loss and screening time; while shortcoming of open reduction of fracture in comparison with DHS (*p* < 0.05). No significant difference was found in the items of required blood transfusion and mean surgical time between IMN and DHS (p ≥ 0.05).

**Fig. 7:**
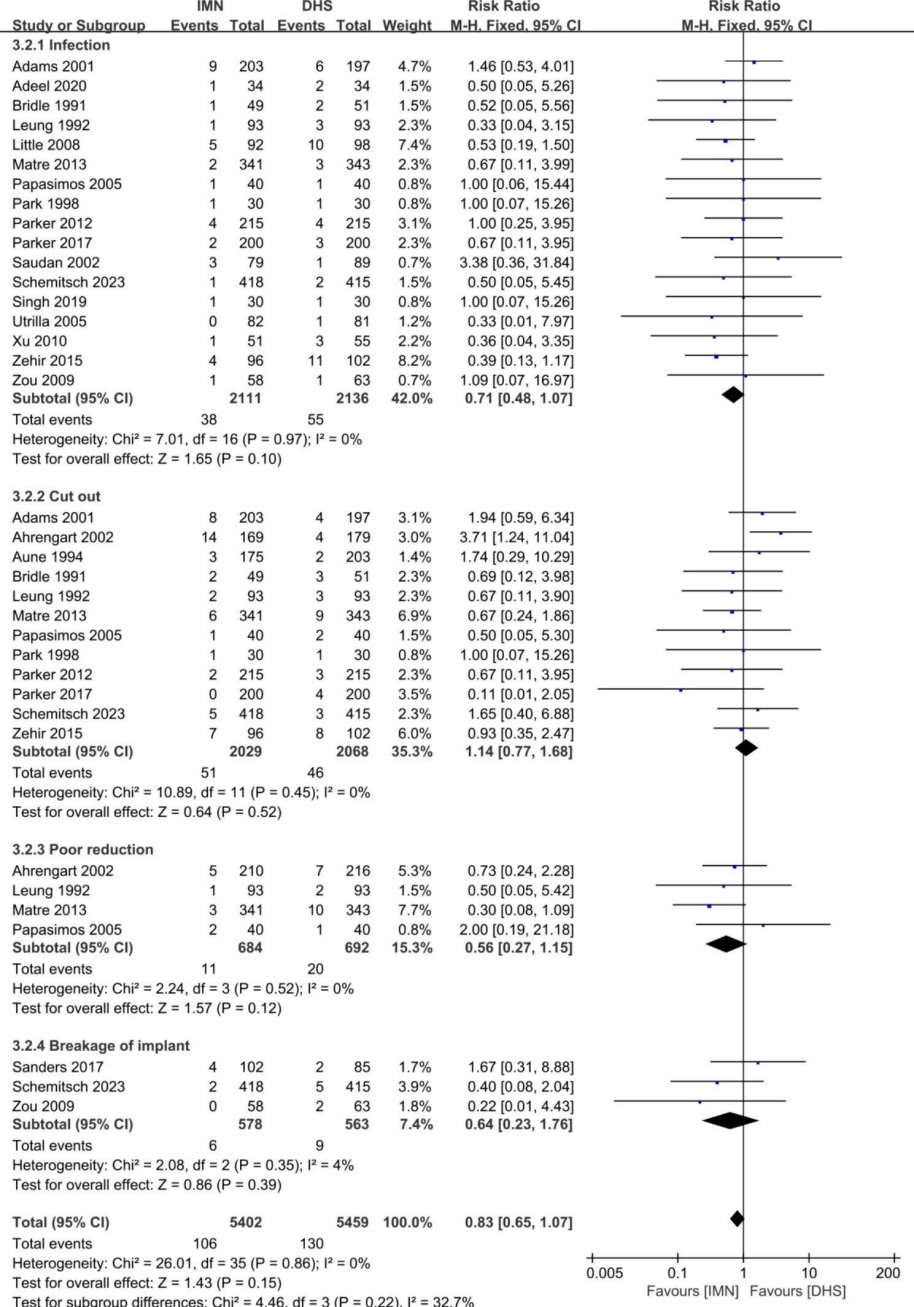
Forest plot comparing the complications after subgroup analysis of infection, cut out, poor reduction and breakage of implant

**Fig. 8:**
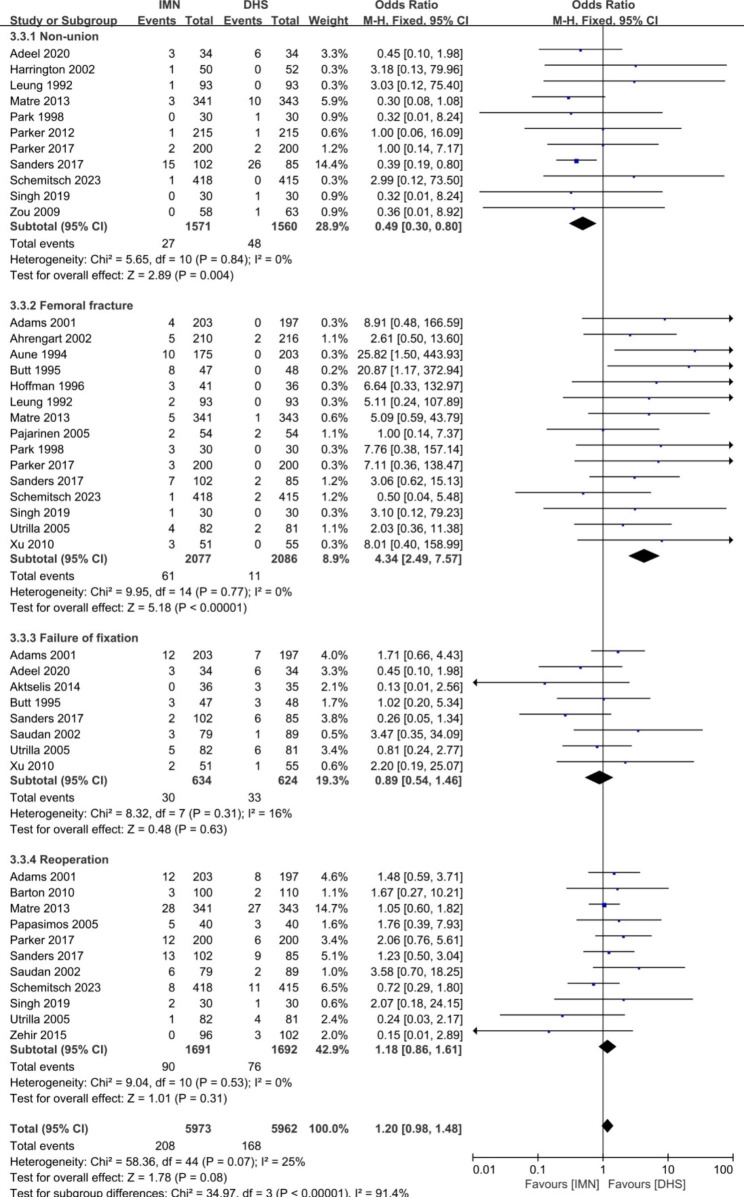
Forest plot comparing the complications after subgroup analysis of non-union, femoral fracture, failure of fixation and reoperation

**Fig. 9:**
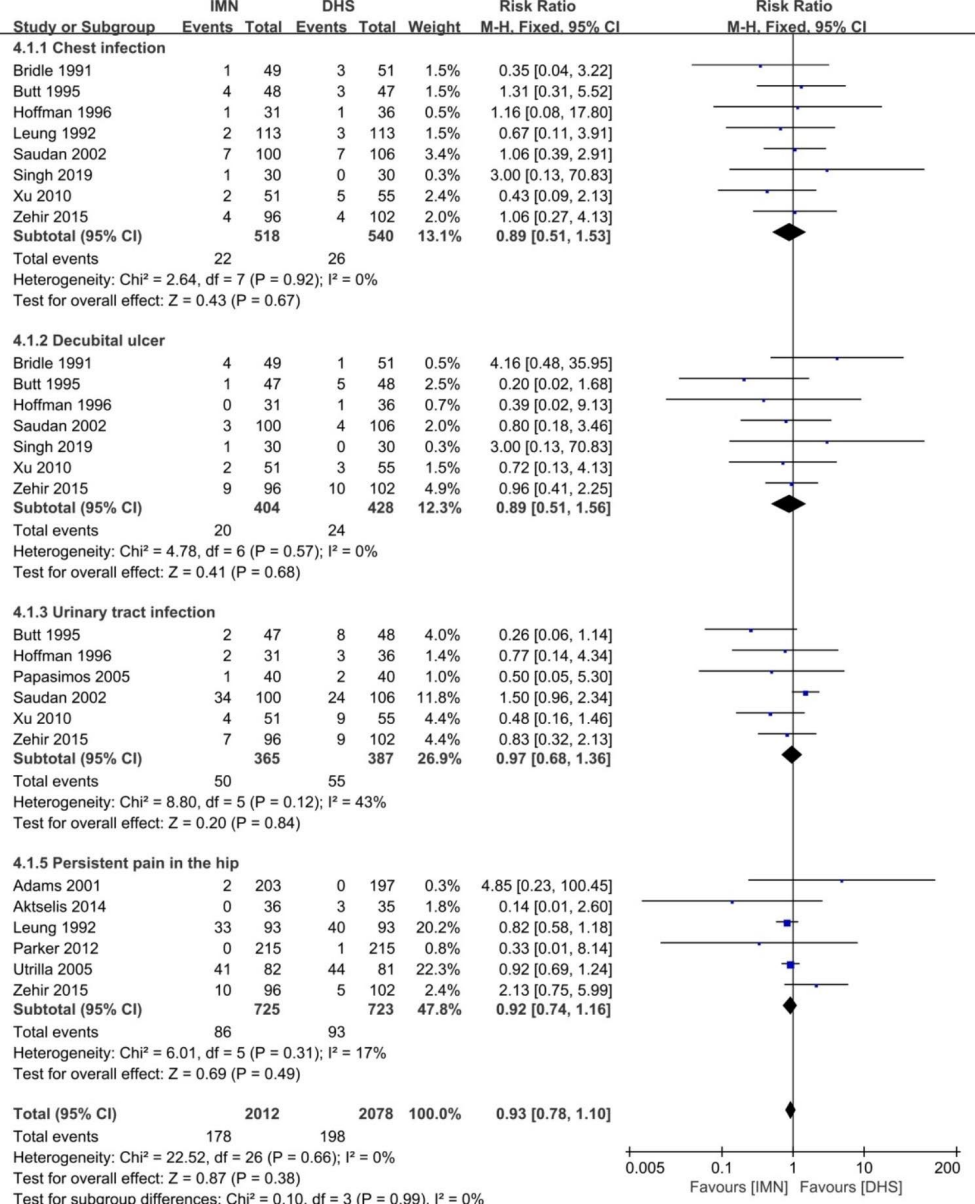
Forest plot comparing the systemic complications of chest infection, decubital ulcer, urinary tract infection and persistent pain in the hip

#### Postoperative details

The postoperative difference between the groups of IMN and DHS was presented in the Fig. 5. There was no significant difference in the items of hospital stays (MD=-0.16 days, 95%CI [-0.63, 0.31], *p* = 0.49), and time to healing (MD=-5.15 days, 95%CI [-29.32, 19.03], p = 0.68), and mean Harris score (MD=-1.02, 95%CI [-12.12, 10.08], *p* = 0.86). Significant difference was found in the parameters of femoral neck shortening (MD=-0.65, 95%CI [-0.96, -0.34], *p* < 0.0001), and mobility score at last follow-up (MD = 0.29, 95%CI [0.01, 0.57], *p* = 0.04). There was heterogeneity for the parameter of mobility score at last follow-up (*p* = 0.02, I^2^ = 66%). So, the sensitivity analysis was conducted by excluding the study of Xu et al. [25], and then the remaining studies were homogeneous (*p* = 0.22, I^2^ = 31%). No significant difference was found in the parameters of mobility score at last follow-up (95%CI [-0.06, 0.38], *p* = 0.15). For other parameters of femoral neck shortening, time to healing, and mean Harris score, the heterogeneity was inevitable between the studies after sensitivity analysis, and the random-effect model was applied to pool the data for statistical analysis. The results of post-operative details showed that there were advantages of IMN in the items of preventing femoral neck shortening (*p* < 0.05). No significant difference was found in the items of hospital stays, time to healing, mean Harris score and mobility score at last follow-up between IMN and DHS (p ≥ 0.05).

**Table 2:** The pooled data for the complications of included studies

Complications	IMN			DHS		Z value	P value
	N/Total	%		N/Total	%		
Infection	38/2111	1.80%		55/2136	2.57%	1.65	0.10
Cut out	51/2029	2.51%		46/2068	2.22%	0.64	0.52
Poor reduction	11/684	1.61%		20/692	2.89%	1.57	0.12
Breakage of implant	6/578	1.03%		9/563	1.60%	0.86	0.39
Non-union	27/1571	1.72%		48/1560	3.1%	3.07	0.004
Femoral fracture	61/2077	2.93%		11/2086	0.50%	5.18	< 0.0001
Failure of fixation	30/634	4.73%		33/624	5.29%	0.48	0.63
Reoperation	90/1691	5.32%		76/1692	4.50%	1.01	0.31
Chest infection	22/518	4.24%		26/540	4.81%	0.43	0.67
Decubitus ulcer	20/404	5.0%		24/428	5.61%	0.41	0.68
Urinary tract infection	50/365	13.7%		55/387	14.2%	0.20	0.84
Persistent pain	86/725	11.9%		93/723	12.9%	0.69	0.49

#### Complications

The orthopaedic complications between the groups of IMN and DHS were shown in the Fig. 6. The pooled risk ratio showed no significant difference in the items of total orthopaedic complications (OR = 1.36, 95%CI [0.94, 1.97], *p* = 0.11). Heterogeneity was found for the pooled 19 studies (*p* = 0.0002, I^2^ = 62%). The subgroup analysis for orthopaedic complications was used to further explore the source of heterogeneity (Figs. 7 and 8). No heterogeneity was found in the parameters of infection, cut out, poor reduction, breakage of implant, nonunion, femoral fracture, failure of fixation, and reoperation rate (p ≥ 0.05, I^2^ < 50%), and the fixed-effect model was used to merge the data. Significant difference between two groups was found in the parameters of non-union (OR = 0.49, 95%CI [0.30, 0.80], p = 0.004), and femoral fracture (OR = 4.34, 95%CI [2.49, 7.57], p < 0.0001). There was no significant difference in the items of infection, poor reduction, breakage of implant, failure of fixation, and reoperation (*p* ≥ 0.05). The pooled data of each complication rate was presented in Table 2.

#### Systemic complications

The difference of systemic complications between two groups was presented in the Fig. 9. There was no significant difference in the items of chest infection (OR = 0.89, 95%CI [0.51, 1.53], p = 0.67), decubitus ulcer (OR = 0.89, 95%CI [0.51, 1.56], p = 0.68), urinary tract infection (OR = 0.97, 95%CI [0.68, 1.36], p = 0.84), and persistent pain in the hip (OR = 0.93, 95%CI [0.78, 1.10], p = 0.49). No heterogeneity was found between the studies (p ≥ 0.05, I^2^ < 50%), and the fixed-effect model was applied to pool the data.

## Discussions

Hip fractures are becoming a social concern problem with aging. These fractures bring a huge economic burden on healthcare care system because of extended hospital stays, co-morbidity and mortality [2, 26]. The goal of internal fixation for hip fractures was to achieve timely healing, early mobility, optimal functional outcome and less complication. The protocol of surgical treatment is becoming the priority choice for hip fractures on account of the advantages of early rehabilitation and activity. The IMN and the DHS are both recommended surgical procedures for intertrochanteric fractures. Treatment decisions are often guided by the surgeon’s own preference and the stability of the fracture. Nevertheless, there are conflicting findings concerning outcomes and postoperative results in the literature [26–28]. For the femoral neck fracture in elderly patients with a displaced fracture in coxa vara, arthroplasty will be preferred, whereas for non-displaced or coxa-valga fractures, as well as in younger patients, osteosynthesis will be performed [29]. In present study, we conducted a comparative analysis to explore the difference in the process of intra-operation, post-operation and total complications between two groups of IMN versus DHS.

Our meta-analysis revealed that significant difference between IMN and DHS was found in terms of open reduction of fracture, blood loss, screening time, femoral neck shortening and the complications of non-union and femoral fracture. No significant difference was found in other parameters of required blood transfusion, mean surgical time, hospital stays, time to healing, mean Harris Hip Score, Mobility Score, infection, cut out, poor reduction, breakage of implant, failure of fixation, and reoperation, and systemic complications of chest infection, decubital ulcer, urinary tract infection and persistent pain in the hip. The results were different from the meta-analysis from Zhang (2023) [18] and Wessels (2022) [19] for the reasons of RCT and Non-RCT studies included for their studies to pool the data. In present study, it was easy to understand that open reduction of fracture, less blood loss in operation, and need more screening time for the group of IMN. The operation of IMN required surgeons to use minimally invasive technique, then reducing open reduction of fracture, blood loss in operation and needing more fluoroscopy time in the process of intra-operation. Our meta-analysis showed that mean blood loss of IMN group was about 124.43ml (95% CI: -169.76, -79.09) less than that in DHS group. A meta-analysis based on 5 randomized controlled trials (RCTs) also proved that less blood loss (p < 0.0001) was found in the PFN group in comparison with the DHS group [30]. Mean femoral neck shortening in IMN group was 0.65 mm (95% CI: -0.96, -0.34) less than that DHS group. The explanation was that the advantage of IMN design was to produce the secondary stability when the failure of initial stability occurred [8, 12]. However, only 2 studies of 30 included papers gave an introduction of the data of femoral neck shortening, and the heterogeneity was presented after the pooled data for the 2 studies. The accuracy of the results need more randomized control studies to confirm. No difference was found in the functional outcome of mean Harris scores and the mobility scores between two groups. It was revealed that the function of hip was similar when hip fracture treated either IMN or DHS during more than 4 months follow-up. Matre et al. [31] and Parker et al. [32] also found no differences in functional outcomes using Parker Mobility Score and EQ-5D. However, the accuracy of the results need more randomized control studies to confirm for the reason of heterogeneity. The rate of non-union for IMN group (27/1571) was less common than that for DHS group 48/1560) with the OR = 0.49 (95% CI: 0.30, 0.80). The higher union rate for IMN group may be attributed to the biomechanical advantage (smaller offset) and secondary stability design for IMN [8, 9, 12]. However, the rate of femoral fracture for IMN group (61/2077) was more common than that of DHS groups (11/2086) with the OR = 4.34 (95% CI: 2.49, 7.57). Femoral fracture may be related to the insertion of main nail [33–36], the process of distal locking [34, 37], and during reaming [38]. The complication of “iatrogenic fracture” in intra-operation should be stressed in the treatment of hip fractures using IMN. It is suggested that special attention should be paid to the risk of femoral fracture when intramedullary nail was inserted in the intraoperative.

Our meta-analysis showed no significant difference was found between two groups in the complications of infection, cut out, poor reduction, breakage of implant, failure of fixation, and reoperation. In current studies, 17 RCT studies were pooled to analyze the complication of infection. 38 cases (1.8%) in the IMN groups and 55 cases (2.57%) in the DHS group developed infection, no difference was found between two groups (*p* > 0.05). Poor bone quality, loss of reduction, excessive collapse, and cut out are frequent causes for failure of fixation in treatment of these fractures [8, 15]. In present study, 12 included studies reported the rate of cut out between two groups. The complication rate of cut out for IMN group was 2.51% (51/2029) similar to that for DHS groups (2.22%, 46/2068). Many authors have reported high reoperation rates with DHS for hip fractures, and the most common causes of failure are screw cut-out and fracture collapse [8, 12, 39]. In present study, no significant difference was found in term of cut out between two groups (p = 0.87). Our meta-analysis also proved no significant difference in the aspect of failure of fixation and reoperation. Yu et al. [40] reported that the reoperation rate of 6.4% for PFNA and of 13.4% for DHS groups. After the pooled data for 11 included studies, the reoperation rates were 5.32% (90/1691) for IMN group and 4.50% (76/1692) for DHS groups. Systemic complications were also analyzed using meta-analysis. No significant difference was found in terms of chest infection, decubitus ulcers, urinary tract infection and persistent pain in the hip. The most complications were the urinary tract infection (13.7% for IMN group and 14.2% for DHS group), then persistent pain in the hip (11.9% for IMN group and 12.9% for DHS group) after the data pooled for included studies (Table 2). It was indicated that the systemic complications of urinary tract infection and persistent pain (over 10%) should be stressed when hip fracture after operation.

Compared with previous meta-analysis [18–20], the merit was that only 30 RCT studies rather than RCT and non-RCT were included for meta-analysis, and the main outcomes in intraoperative and postoperative details were investigated. The limitations were listed as follows. First, the quality of this meta-analysis was limited by the quality of available literatures. Second, the difference in the implant design was applied across the studies. In present studies, we considered the IMN devices including PFNA, PFN, INTERTAN nail, and GN as the same type of internal fixation device for hip fractures. However, the Cochrane review on IMNs demonstrated no difference in the complications [41]. Third, some studies included were adjudged to have a moderate or higher overall risk of bias, largely due to the lack of study protocols, which increases the risk of reporting and measurement biases.

## Conclusions

Based on the results, our meta-analysis revealed that hip fractures treated with IMN have advantages of blood loss, prevention of femoral neck shortening, and the rate of non-union, with shortcoming of open reduction, screening time and femoral fractures. As more and more surgeons are choosing intramedullary fixation for the treatment of hip fracture, we recommend special attention should be paid to the risk of femoral fracture when IMN was inserted.

## Data Availability

The datasets used and/or analyzed during the current study are available from the corresponding author on reasonable request.

## Abbreviations

DHS: Dynamic hip screw
IMN: Intramedullary nail
Targon PF: Targon Proximal Femoral Nail
GN: Gamma Nail
PFN: Proximal Femoral Nail
PFNA: Proximal Femoral Nail with Anti-rotation
RCT: Randomized Controlled Trials
SMD: Standardized mean differences
95%CI: 95% confidence intervals
OR: Odds ratio
